# The income-happiness nexus: uncovering the importance of social comparison processes in subjective wellbeing

**DOI:** 10.3389/fpsyg.2023.1283601

**Published:** 2023-11-23

**Authors:** Pål Kraft, Brage Kraft

**Affiliations:** ^1^Department of Psychology, University of Oslo, Oslo, Norway; ^2^Department of Psychology, Oslo New University College, Oslo, Norway; ^3^Department of Behavioural Sciences, Oslo Metropolitan University, Oslo, Norway

**Keywords:** income, education, subjective socioeconomic status, social comparison, subjective wellbeing, mediation

## Abstract

**Introduction:**

Previous research has established a positive correlation between income and subjective wellbeing (SWB). This correlation is attributed to income’s ability to provide material circumstances and influence one’s perceived socioeconomic rank in society, known as subjective socioeconomic status (SES).

**Objective:**

This study sought to examine whether social comparison processes could mediate the relationship between income and SWB. Specifically, we aimed to explore the impact of comparing one’s current socioeconomic situation to individuals from a similar socioeconomic background (referred to as Comsim) on SWB, based on the similarity hypothesis of social comparison theory.

**Methods:**

Data stem from two separate two-wave surveys. Study 1 comprised 588 participants, with 294 men and 294 women; age range 25–60 years; mean age 41.5 years). Study 2 comprised 614 participants, with 312 men and 302 women; age range 25–60 years; mean age 43.5 years. In both studies, data on predictors and SWB were collected 3 months apart.

**Results:**

In both study 1 and study 2, bivariate analysis demonstrated a positive correlation between income and SWB. However, multivariate regression models revealed that income did not have a direct effect on SWB. Instead, in both studies, subjective SES and Comsim emerged as significant predictors of SWB, with Comsim being the most influential. Furthermore, our formal mediation analysis indicated that subjective SES and Comsim fully mediated the relationship between income and SWB, when combined. Additionally, in study 2, we found that cognitive factors such as personal control, as well as affective factors like self-esteem, played a mediating role between the social comparison processes and SWB.

**Conclusion:**

This study contributes to existing research by emphasizing the importance of two distinct social comparison mechanisms in mediating the relationship between income and SWB.

**Implications:**

Therapeutic interventions to improve SWB should also consider social comparison processes. From a political standpoint, policies addressing income inequality can mitigate the negative effects of social comparisons on wellbeing. Providing support to those in lower socioeconomic positions can also enhance SWB.

## 1 Introduction

Subjective wellbeing (SWB) encompasses an individuals overall assessment and emotional experiences in relation to their life (Diener et al., 1985). SWB is a tripartite model consisting of three main components: life satisfaction, positive affect (positive emotions), and negative affect (negative emotions). SWB comprises two distinct components: cognitive wellbeing and affective wellbeing (Diener, 1984; Luhmann et al., 2012b). Cognitive wellbeing involves the cognitive evaluation of one’s overall life satisfaction (Diener et al., 2010), while affective wellbeing focuses on the frequency and intensity of positive and negative emotions and mood (Luhmann et al., 2012a). These elements together provide a comprehensive understanding of an individual’s SWB (Diener, 1984). There are two important approaches to wellbeing: eudaimonic wellbeing, which focuses on discovering genuine human potential, meaning in life, and engagement with life’s challenges; and hedonic wellbeing, which emphasizes experiencing pleasure and avoiding pain (Ryan and Deci, 2001). Subjective wellbeing is based on the hedonic school of wellbeing.

Among the most critical predictors of SWB are individuals’ social relationships, including the quality of their interactions and support networks, personality traits, such as extroversion and neuroticism (Richard and Diener, 2009), and their physical and mental health (Ngamaba et al., 2017). Additionally, income has been identified as an important predictor of SWB. Hence, a positive association between income and SWB has been extensively documented in the literature (Howell and Howell, 2008; Diener et al., 2018; Tan et al., 2020; Killingsworth et al., 2023). However, the mechanism underlying this relationship is still not fully understood (Diener et al., 2018; Tan et al., 2020). A materialist explanation emphasizes that the lack of money may hinder individuals from meeting their basic needs and attaining optimal living conditions (Diener et al., 1993; Diener and Oishi, 2000; Shah et al., 2012; Haushofer and Fehr, 2014). This can have adverse effects on SWB by limiting access to healthcare, leisure activities, desirable goods, and other benefits (George, 1992; Shah et al., 2012; Haushofer and Fehr, 2014; Diener et al., 2018). Additionally, negative living conditions such as economic worries, job insecurity, substandard housing, overcrowding, pollution, noise, heavy traffic, and neighborhood crime can be associated with lower SWB (Steptoe and Marmot, 2003; Evans and Kim, 2007; Thoits, 2010; Blane et al., 2013; McGowan and Shahab, 2019). According to this perspective, increasing actual income should lead to improved SWB, particularly for disadvantaged individuals (Marmot, 2005; Shah et al., 2012; Haushofer and Fehr, 2014; Nettle, 2021; McGuire et al., 2022). However, recent research has challenged the ability of the materialist explanation to fully account for the relationship between income and SWB. For instance, studies have demonstrated that income matters for SWB across the entire income distribution, not just at the lower end (Tan et al., 2020; Killingsworth, 2021). Notably, the positive correlation between income and SWB is evident even within high-income groups such as millionaires (Donnelly et al., 2018).

The relativity hypothesis proposes that an individual’s subjective position in the socioeconomic hierarchy, known as subjective socioeconomic status (subjective SES), has a significant impact on their SWB (Howell and Howell, 2008; Lucas and Schimmack, 2009; Diener et al., 2018; Tan et al., 2020; La et al., 2021). Subjective SES refers to an individual’s perception or self-assessment of their own social and economic standing within a society (Adler et al., 1994). Unlike objective socioeconomic status, which is typically determined by objective indicators like income, education, and occupation, subjective socioeconomic status is based on an individual’s personal feelings and beliefs about their position in relation to others. This subjective assessment can be influenced by various factors, including cultural background, life experiences, and social comparisons, and it may not always align with objective measures of socioeconomic status. It is often used in research to understand how people’s perceptions of their social and economic status relate to various outcomes, including health and SWB.

While subjective SES is recognized as a crucial mediator between income and SWB, it is important to note that subjective SES influences SWB beyond its role as a mere mediator of income (Tan et al., 2020). In fact, subjective SES has been found to be a stronger predictor of SWB than actual income in multivariate models. Two primary mechanisms have been proposed to explain the impacts of subjective SES on SWB (Adler et al., 2000; Operario et al., 2004; Euteneuer, 2014; Quon and McGrath, 2014; Präg et al., 2016; Demakakos et al., 2018; Tan et al., 2020; Vezzoli et al., 2022). The first mechanism suggests that a lower subjective SES is associated with an increased perception of unfair social inequality and personal relative deprivation. Personal relative deprivation refers to an individual’s perception that they are unfairly worse off or have less than others with whom they compare themselves, whether in terms of income, opportunities, or other resources. It is a subjective feeling of being disadvantaged or deprived in comparison to a reference group. This perception of relative disadvantage can negatively affect SWB by triggering feelings of anger, frustration and other negative emotions (Gallo and Matthews, 2003; Matthews et al., 2010; Matthews and Gallo, 2011; Callan et al., 2015; Kim et al., 2017). The second proposed mechanism of subjective SES resolves around the effects of social comparison. Social comparison is a psychological process where individuals evaluate themselves by comparing their own abilities, attributes, or circumstances to those of others (Festinger, 1954; Buunk and Gibbons, 2007). This comparative analysis can occur in various aspects of life, such as appearance, wealth, achievements, or social status. Social comparisons can be either upward, where individuals compare themselves to those they perceive as superior, or downward, comparing to those they see as inferior. This process can have both positive and negative effects (Smith, 2000; Buunk and Gibbons, 2007; Dufhues et al., 2023). It can motivate personal improvement and provide a sense of belonging when the comparisons are favorable, but it can also lead to feelings of inadequacy, jealousy, and low self-esteem when comparisons are unfavorable. Social comparison plays a significant role in shaping self-perception, social interactions, and overall wellbeing.

Downward comparison, where an individual compares themselves to someone they perceive as inferior in the social hierarchy, usually leads to improved mood and positive self-evaluations (Collins, 1996; Buunk and Gibbons, 2006, 2007). Conversely, upward comparison, which is more commonly observed, can lead to negative emotions and negative self-evaluations (Wheeler and Miyake, 1992; Buunk and Gibbons, 2006, 2007). The similarity hypothesis is a fundamental concept within the framework of Social Comparison Theory (Festinger, 1954). This hypothesis suggests that individuals are more likely to engage in social comparisons with others who are similar to themselves in relevant aspects. In other words, people tend to compare themselves with those who share similar characteristics, such as age, background, or social status. The underlying idea is that comparing oneself to someone who is similar allows for a more accurate assessment of one’s abilities, traits, or circumstances. It also minimizes the potential for threatening or distressing comparisons that might occur when comparing with someone significantly different or superior. The similarity hypothesis helps explain how individuals seek out relevant reference points for self-evaluation and how these comparisons can influence their self-esteem and behaviors. It highlights the role of social context and the selection of comparison targets in shaping our perceptions and reactions in various life domains (Festinger, 1954; Buunk and Gibbons, 2007; Clark and Senik, 2010; Van Praag, 2011).

Hence, in this study, we examine whether comparing oneself socioeconomically to similar others (Comsim) predicted SWB beyond income and subjective SES. Comsim was operationalized as comparing one’s current socioeconomic to that of childhood friends and schoolmates. Additionally, we explore the extent to which subjective SES and Comsim act as mediators in the relationship between income and SWB. In our second study, we aim to replicate the outcomes of Study 1 using alternative measures of SWB. Furthermore, Study 2 includes perceived social mobility as a covariate in predicting SWB, as recent research has identified it as a significant predictor of SWB (Euteneuer and Schäfer, 2018; Mendoza et al., 2018; Yan et al., 2018; Gugushvili et al., 2019, 2022; Tan et al., 2020; Euteneuer et al., 2021; Gugushvili, 2021; Gugushvili and Präg, 2021; Präg and Gugushvili, 2021). Finally, Study 2 explores whether cognitive (personal control) and affective factors (self-esteem) mediate the relationship between social comparison processes (subjective SES and Comsim) and SWB. This expectation is based on previous research that has established correlations between personal control (Bandura, 2001; Barlow et al., 2002; Bandura et al., 2003; Kuijer and De Ridder, 2003; Leganger and Kraft, 2003; Santos, 2014; Kraft et al., 2022), self-esteem (Diener and Diener, 1995; Seligman and Csikszentmihalyi, 2000; Robins et al., 2001; Twenge and Campbell, 2002; Baumeister et al., 2003; Tesser, 2003; Diener et al., 2015), socioeconomic status and SWB.

### 1.1 Hypotheses

**FIGURE 1:**
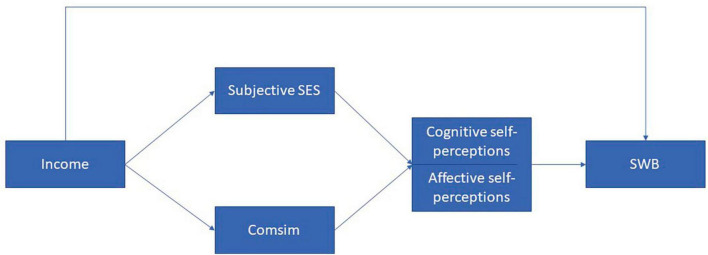
Theoretical model in which social comparison and self-perceptions partially mediate the relationship between income and subjective wellbeing (SWB). Comsim = comparing oneself with similar others.

Figure 1 provides a visual representation of the theoretical model that serves as the guiding framework for the hypotheses. Hypotheses I and II replicate from previous research, establishing a foundation for hypotheses III-V. These latter hypotheses introduce innovative perspectives and contribute new insights to the field. Our study’s specific hypotheses are as follows:

I. Income is associated with SWB across the entire income distribution.
II. Subjective SES predicts SWB independently of income.
III. Comparing oneself to similar others (Comsim) predicts SWB independently of subjective SES.
IV. Subjective SES and Comsim jointly mediate the relationship between income and SWB.
V. The association between subjective SES and Comsim and SWB is mediated by personal control and self-esteem.

## 2 Study 1

### 2.1 Research design

We implemented a two-wave survey design to collect data on gender, age, education, income, subjective SES, and Comsim at time 1 (T1). Subsequently, 3 months later, data on SWB were collected at T2. Participants accessed an online Qualtrics survey by following a provided link in the study invitation at T1. Additionally, all T1 participants were contacted via email and provided with a link to the second Qualtrics survey at T2.

### 2.2 Sampling procedure

Participants were recruited via Prolific, an online research platform known for ensuring data quality (Peer et al., 2017; Palan and Schitter, 2018). Detailed information regarding Prolific’s sampling process can be located in the Supplementary material.^1^ Eligible participants meeting the following criteria were invited to take part in the study: age 25–60 years (as individuals typically complete their education by around the age of 25), possessing UK/British citizenship (to ensure consistent reporting of education), and having English as their first language (to ensure sufficient comprehension of the study questions). The data collection took place in February (T1) and May 2022 (T2).

### 2.3 Participants

In accordance with existing reviews and meta-analyses, we anticipated observing a positive association between income and SWB, with an effect size ranging from 0.15 to 0.25. Moreover, we expected to find small to moderate effect sizes for the mediating variables (Kraus et al., 2009; Tan et al., 2020). To ensure sufficient power (i.e., 8 power) to detect even a small mediation effect and taking into consideration an estimated 75% retention of participants from T1 to T2 (Cho and Lumsden, 2023), our goal was to recruit a minimum of 670 participants at T1, with a balanced representation of genders.

Recruitment of participants was concluded after enrolling a total of 679 participants at T1, with 339 men and 340 women. At T2, the final sample comprised 588 participants, consisting of 294 women and 294 men. The average age of the participants was 41.5 years (SD = 9.9 years), indicating an 86.6% retention rate.

### 2.4 Measures

#### 2.4.1 SWB

Cognitive wellbeing was assessed using the Satisfaction with Life Scale (SWLS) (Diener et al., 1985). Participants were presented with five statements and asked to rate their agreement on a 7-point scale ranging from strongly agree to strongly disagree. An example from the SWLS is: “If I could live my life over, I would change almost nothing.” Participants responses to the five items were summed and averaged, with higher values reflecting greater satisfaction with life (Cronbach’s α = 0.93).

Affective wellbeing was assessed using the Centre for Epidemiological Studies Depression Scale (CES-D) (Radloff, 1977). This 20-item instrument captures a range of emotions including positive and negative affect, as well as feelings of guilt and worthlessness. Participants were asked to indicate the frequency with which they experienced each item over the past week, using a 4-point scale ranging from rarely or none of the time (less than 1 day) to most or all of the time (5–7 days). An example item from the CES-D is: “I felt sad.” Participants responses were summed and averaged, with higher values indicating higher levels of negative affect and depressed mood (Cronbach’s α = 0.93).

#### 2.4.2 Income

Participants’ income was determined using the following question: “What is your income per year (after tax) in GPB?” They were presented with twelve options, spanning from less than £10,000 to £150,000 or more. To ensure a balanced distribution of variables, income was recoded into six categories: (1) less than £10,000 (*n* = 131), (2) £10,000–£19,999 (*n* = 129), (3) £20,000–£29,999 (*n* = 151), (4) £30,000–£39,999 (*n* = 93), (5) £40,000–£49,999 (*n* = 55), and (6) £50,000 or more (*n* = 29).

#### 2.4.3 Education

Participants’ education level was determined using the following question: “Which of these is the highest level of education you have completed?” They were presented with twelve options, spanning from no formal qualifications to a doctorate degree. Education was recoded into five groups: (1) no formal education/secondary education (e.g., GED/GCSE) (*n* = 93), (2) high school diploma/A-levels (*n* = 103), (3) technical/community college (*n* = 71), (4) undergraduate degree (BA/BSc/other) (*n* = 223), and (5) graduate degree (MA/MSc/MPhil/other)/doctoral degree (*n* = 98).

#### 2.4.4 Subjective SES

Subjective socioeconomic status (SES) was assessed using the MacArthur Scale of Subjective Social Status–Adult Version developed by Adler et al. (1994). Participants were presented with a visual representation of a ladder consisting of ten rungs. The ladder was accompanied by a descriptive text that explained its meaning: “This ladder represents where people stand in society. At the top of the ladder are people who are the best-off, those who have the most money, most education, and best jobs. At the bottom are people who are the worst-off, those who have the least money, least education, worst jobs, or no job. Please place an ‘X’ on the rung that best represents where you think you stand on the ladder.” Participants indicated their subjective position by marking an ‘X’ on the ladder, with scores ranging from 1 (lowest rung) to 10 (highest rung).

#### 2.4.5 Comsim

For the purpose of this study, a four-item instrument was specifically developed to measure comparison to similar others (Comsim). Each item began with the statement “Compared to other people coming from a similar socioeconomic background” and encompassed the following statements: (1) “My current financial situation is quite good”; (2) “My current educational situation is quite good”; (3) “I have been quite successful in work-life”; and (4) “I think my current socioeconomic position is quite good.” Participants responded to these items using a 7-point scale ranging from strongly disagree to strongly agree.

To assess the psychometric properties of the Comsim instrument, a principal component factor analysis with varimax rotation was conducted on the four items. The analysis yielded a one-factor solution that accounted for 78.8% of the inter-item variance (Eigenvalue = 3.15). The internal consistency reliability of the scale was assessed using Cronbach’s alpha, which resulted in a coefficient of 0.91, indicating high internal consistency. Consequently, the Comsim scale consolidates the mean of the four items into a single variable.

For further details on this variable, please refer to Table 1 and the Supplementary material (see text footnote 1) for a comprehensive description.

**TABLE 1:** Descriptive statistics Study 1 (*N* = 588).

Variables	*M*	SD	α
Age	41.53	9.94	
Education	4.24	1.39	
Income	2.93	1.71	
Subjective SES	5.21	1.65	
Comsim	4.38	1.43	0.91
CES-D	1.90	0.59	0.93
SWLS	4.10	1.50	0.93

*M*, mean; Std, standard deviation; α, Cronbach’s α; CES-D, center for epidemiological studies depression scale; SWLS, satisfaction with life scale.

### 2.5 Data analysis

The statistical analyses involved several steps. Initially, descriptive statistics were examined to provide an overview of the variables, followed by the calculation of bivariate correlations to explore the relationships between the variables. To ensure comparability and facilitate analysis, the variables were standardized. Next, a series of linear multiple regression analyses were conducted to test hypotheses II-IV. SWB was the dependent variable, while income, subjective SES, and Comsim served as independent variables. Additionally, the models included gender, age, and education as covariates to account for their potential influence on SWB. These regression analyses were performed using IBM SPSS version 29. To investigate hypothesis V regarding multiple mediation, the PROCESS macro (model 4) developed by Hayes (2022) was utilized. This approach enabled the assessment of the mediating effects of subjective SES and Comsim on the relationship between income and SWB. To evaluate the reliability of the findings, bootstrapping with 5,000 samples was employed. Bootstrapping provides robust estimates of indirect effects and helps determine the significance of the mediation effects.

### 2.6 Results

No statistically significant differences were found in demographic variables between participants who completed both T1 and T2, and those who dropped out at T2. Detailed analyses of these results can be accessed in the Supplementary material (see text footnote 1). Internal consistency was acceptable for Comsim, CES-D, and SWLS. Descriptive statistics for the variables are presented in Table 1.

Table 2 displays the correlations between the study variables. The results confirm the anticipated association between income and the two measures of SWB. To provide a comprehensive understanding of this association, supplementary analyses were conducted by categorizing income into low, medium, and high groups. The lowest income group exhibited an average CES-D score of 2.04, while the highest income group had an average score of 1.68, indicating a difference of 0.6 standard deviations. Similarly, the mean SWLS scores for the lowest and highest income groups were 3.51 and 4.88, respectively, reflecting a difference of 0.9 standard deviations.

**TABLE 2:** Pearson’s correlation (r) between variables (*N* = 588).

Variables	1	2	3	4	5	6
1. Education		0.24**	0.30**	0.17**	-0.08*	0.10*
2. Income			0.44**	0.39**	-0.17**	0.24**
3. Subjective SES				0.56**	-0.36**	0.46**
4. Comsim					-0.43**	0.64**
5. CES-D						-0.53**
6. SWLS						

*p < 0.05 (two-tailed tests); **p < 0.01 (two-tailed tests).

CES-D, center for epidemiological studies depression scale; SWLS, satisfaction with life scale.

Furthermore, there was a moderate, positive correlation between income and education. Education, on the other hand, displayed only weak associations with CES-D and SWLS. Subjective SES and Comsim demonstrated a strong positive correlation. Both subjective SES and Comsim exhibited substantial correlations with CES-D and SWLS in the expected directions, providing further support for the study’s hypotheses.

The results of the regression analyses are presented in Table 3. Model 1, which controlled for gender, age, and education, revealed that income significantly predicted CES-D and SWLS, while education did not predict SWB in this model. However, after adding subjective SES as a predictor in Model 2, income became insignificant while subjective SES emerged as a significant predictor of CES-D and SWLS. The inclusion of subjective SES led to a significant increase in the variance explained (Δ*F* = 61.77 and 11.82, respectively, both *p* < 0.001).

**TABLE 3:** Hierarchical regressions of associations between predictors and SWB (beta-coefficients) (*N* = 588).

	Dependent variables	
Predictors	CES-D	SWLS
**Model 1**		
Gender	-0.02	0.10*
Age	-0.18**	-0.02
Education	-0.06	0.04
Income	-0.16**	0.25**
*R* ^2^	0.06	0.07
Δ*R*^2^	0.06	0.07
Δ*F*	9.74	11.15
**Model 2**		
Gender	0.00	0.07
Age	-0.16**	-0.05
Education	0.01	-0.05
Income	-0.02	0.07
Subjective SES	-0.34**	0.44
*R* ^2^	0.14	0.22
Δ*R*^2^	0.08	0.14
Δ*F*	61.77	11.82
**Model 3**		
Gender	0.03	0.02
Age	-0.14**	-0.08*
Education	0.01	-0.05
Income	0.05	-0.04
Subjective SES	-0.19**	0.18**
Comsim	-0.34**	0.58**
*R* ^2^	0.22	0.44
Δ*R*^2^	0.07	0.22
Δ*F*	55.94	225.25

*p < 0.05, **p < 0.01. SWLS, satisfaction with life scale; CES-D, center for epidemiological studies depression scale.

In Model 3, Comsim was added as an additional predictor. In this model, both subjective SES and Comsim predicted CES-D and SWLS, with Comsim exerting a stronger influence. The inclusion of Comsim resulted in a significant increase in the variance explained (Δ*F* = 55.94 and 225.25, respectively, both *p* < 0.001). These findings suggest that subjective SES and Comsim play important roles in predicting SWB, with Comsim having a greater impact than subjective SES.

**FIGURE 2:**
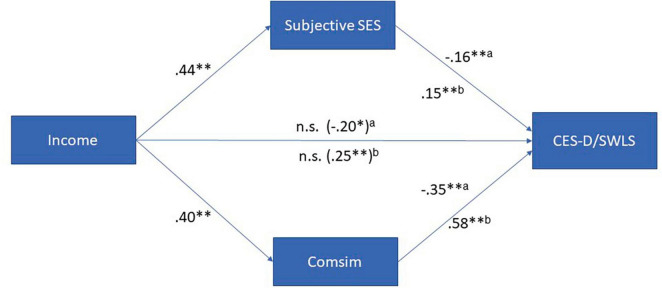
Subjective and comparative SES as mediators of the relationship between income and SWB (*N* = 588). ns, not significant; *p ≤ 0.05; **p ≤ 0.001; ^a^effects on CES-D; ^b^effects on SWLS.

To examine the mediation hypotheses (hypothesis IV) involving subjective SES and Comsim, mediation analyses were conducted. These analyses simultaneously tested the mediation effects of Comsim and subjective SES, while controlling for gender, age, income, and education as covariates. The results of these analyses are presented in Figure 2, and additional details can be found in Supplementary Table 1 (see text footnote 1).

The findings indicated that the relationships between income and CES-D, as well as income and SWLS, were completely mediated by both subjective SES and Comsim. Notably, Comsim emerged as a more significant mediator in the income-SWB relationships compared to subjective SES. These results provide evidence for the mediating role of both subjective SES and Comsim, with Comsim demonstrating a stronger influence in mediating the relationship between income and SWB.

### 2.7 Interim discussion

The findings from Study 1 provide support for all four hypotheses. Firstly, the study confirmed the presence of a positive association between income and SWB, which was observed across the entire income range. This supports hypothesis I and confirm previous research showing that income plays a role in determining SWB, regardless of the income level (Tan et al., 2020). Secondly, subjective SES was found to be a strong predictor of SWB, surpassing the influence of income. This supports hypothesis II, is in accord with previous research (Adler et al., 2000; Operario et al., 2004; Euteneuer, 2014; Quon and McGrath, 2014; Präg et al., 2016; Demakakos et al., 2018; Tan et al., 2020; Vezzoli et al., 2022) and highlights the significance of individuals’ subjective evaluation of their socioeconomic position in relation to their wellbeing. Thirdly, the study demonstrated that comparing oneself to similar others (Comsim) predicts SWB above and beyond the influence of subjective SES. This supports hypothesis III and indicates that social comparisons with individuals from a similar socioeconomic background have a unique impact on SWB. This finding expand on previous research. Finally, the mediation analysis revealed that subjective SES and Comsim jointly and fully mediated the relationships between income and the measures of SWB. Notably, Comsim emerged as the more important mediator, lending support to hypothesis IV. These findings align with the similarity hypothesis of social comparison theory and suggest that comparing oneself to similar others is particularly relevant for SWB. Overall, Study 1 provides robust evidence for the importance of income, subjective SES, and Comsim in understanding SWB. The results highlight the complex interplay between these factors and emphasize the significance of social comparisons with similar others in shaping individuals’ wellbeing.

## 3 Study 2

### 3.1 Research design

Similar to Study 1, Study 2 employed a two-wave survey design, and recruited participants through the online platform Prolific. Inclusion criteria were identical to Study 1. Data on gender, age, education, income, subjective SES, Comsim, social mobility, personal control, and self-esteem were collected at T1, while data on SWB were collected 3 months later at T2. The data collection took place in August (T1) and November 2022 (T2).

### 3.2 Sampling procedure

As in Study 1, participants were recruited via Prolific. Detailed information regarding Prolific’s sampling process can be located in the Supplementary material (see text footnote 1). Eligible participants meeting the following criteria were invited to take part in the study: age 25–60 years (as individuals typically complete their education by around the age of 25), possessing UK/British citizenship (to ensure consistent reporting of education), and having English as their first language (to ensure sufficient comprehension of the study questions).

### 3.3 Participants

Recruitment for the study was successfully completed at T1, resulting in a total of 721 participants, consisting of 362 men and 359 women. However, during the follow-up at T2, 109 participants were lost, yielding an 85.2% retention rate. The final sample at T2 included 614 participants, with 302 women and 312 men, and an average age of 43.53 years (SD = 10.79 years).

### 3.4 Measures

Gender, age, education, income, and subjective SES were measured in the same manner as in Study 1. To balance the distribution of variables, income and education were recorded in a manner consistent with Study 1. Income was categorized into six groups: (1) < £10,000 (*n* = 102), (2) £10,000–£19,999 (*n* = 131), (3) £20,000–£29,999 (*n* = 166), (4) £30,000–£39,999 (*n* = 101), (5) £40,000–£49,999 (*n* = 57), and (6) ≥ £50,000 (*n* = 57). Education was divided into five groups: (1) no formal education/secondary education (e.g., GED/GCSE) (*n* = 65), (2) high school diploma/A-levels (*n* = 103), (3) technical/community college (*n* = 86), (4) undergraduate degree (BA/BSc/other) (*n* = 248), and (5) graduate degree (MA/MSc/MPhil/other)/doctoral degree (*n* = 112).

#### 3.4.1 SWB

In Study 2, SWB was measured using SWLS as described in Study 1, and the Positive and Negative Affect Schedule (PANAS) developed by Watson et al. (1988). The PANAS is a widely utilized measure that captures both positive and negative affective states. The PANAS consists of two 10-item scales that assess participants’ experiences of positive affective states and negative affective states. Participants were instructed to indicate the extent to which they have felt each of these affective states over the past month. Response options ranged from very little (1) to a lot (5). For each scale, the average score was computed. To create a measure of affect balance, the negative affect score was subtracted from the positive affect score. This calculation provides an indication of the balance between positive and negative affect experienced by participants. A higher score on the affect balance measure reflects a greater prevalence of positive affect relative to negative affect (Diener et al., 2018).

#### 3.4.2 Comsim

In Study 2, a modified four-item instrument was developed to measure Comsim, which differed slightly from the scale used in Study 1. The aim was to capture the tendency of individuals to compare their current socioeconomic status with that of their childhood friends and schoolmates. This approach was chosen to encompass social comparisons with individuals who have had direct experiences and close associations within the social system (Zell and Alicke, 2010; Dufhues et al., 2023). It was anticipated that childhood friends and schoolmates would generally come from a similar socioeconomic background as the participants (Malacarne, 2017; Boterman, 2019; Brandén and Bygren, 2022; Zwier and Geven, 2023).

The four items assessing Comsim in Study 2 began with the statement: “Think about the friends and schoolmates you had when you were a child. How do you think you have done in life, when you compare yourself to them?” Participants then rated their agreement with the following statements on a scale ranging from strongly disagree (1) to strongly agree (7): (1) “My education is quite good,” (2) “My work-life has been quite successful,” (3) “My income is quite good,” and (4) “My social status is quite high.” To assess the reliability and validity of the Comsim scale, a principal component factor analysis with varimax rotation was conducted. This analysis yielded a one-factor solution that explained 70.6% of the inter-item variance (Eigenvalue = 2.82), indicating a strong underlying factor. The scale demonstrated strong internal consistency, as evidenced by a Cronbach’s alpha coefficient of 0.86, indicating high reliability. Consequently, the Comsim scale consolidates the mean of the four items into a single variable. For more comprehensive insights into the development and properties of the Comsim scale, including additional details, please refer to the Supplementary material (see text footnote 1).

#### 3.4.3 Social mobility

To measure perceived social mobility in relation to one’s parents, modified versions of the subjective SES (MacArthur) ladder were employed. The procedure for assessing perceived social mobility is described in detail in the Supplementary material (see text footnote 1), along with accompanying descriptive statistics.

In this procedure, participants were first asked to rate the position of their father and mother on the MacArthur ladder when they were at the same age as the participant is currently. The ladder represents the social hierarchy, with the top rung indicating individuals who are the best-off in terms of factors such as wealth, education, and job status, while the bottom rung represents those who are the worst-off in these respects. Participants provided their ratings for both their father and mother separately.

The scores from the ratings of the participant’s father and mother were then averaged to obtain a single value. Next, this average value was subtracted from the participant’s self-reported subjective SES score. This calculation yielded a measure of perceived social mobility vis-a-vis one’s parents, reflecting the perceived change in social position compared to one’s parents.

#### 3.4.4 Self-perceptions

To assess personal control, the Sense of Control Scale developed by Lachman and Weaver (1998a) was utilized. This scale measures the sense of control individuals have over their lives and consists of two dimensions: personal mastery and perceived constraints. Personal mastery is captured by four statements, such as “I can do just about anything I really set my mind to” (Cronbach’s α = 0.87). Perceived constraints are assessed by eight statements, including “Other people determine most of what I can and cannot do.” Participants rated their agreement with each statement on a 7-point scale, ranging from strongly disagree to strongly agree (Cronbach’s α = 0.93). Compound scores for personal mastery and perceived constraints were calculated by summing and averaging the relevant items.

Self-esteem was measured using the widely used Rosenberg Self-Esteem Scale (Rosenberg, 1965). This scale comprises 10 items that assess individuals’ positive and negative feelings about themselves, reflecting their overall self-worth. Participants indicated their level of agreement with each statement on a 4-point scale, ranging from strongly agree to strongly disagree. The total score for the scale was computed by summing and averaging the responses, with higher scores indicating higher levels of self-esteem (Cronbach’s α = 0.86).

### 3.5 Results

Descriptive statistics for the study variables are presented in Table 4, providing an overview of the sample characteristics and the distribution of key variables. No statistically significant differences in demographic variables were observed between participants who completed both T1 and T2 assessments and those who discontinued participation at T2. For a comprehensive analysis of these findings, please refer to the Supplementary material.

**TABLE 4:** Descriptive statistics (*N* = 614).

Variables	*M*	SD	α
Age	43.53	10.79	
Education	3.43	1.32	
Income	3.08	1.50	
Subjective SES	5.21	1.72	
Comsim	4.33	1.28	0.86
Social mobility	0.29	1.69	
Personal mastery	5.13	1.19	0.87
Perceived constraints	3.42	1.30	0.93
Self-esteem	2.86	0.66	0.86
SWLS	4.05	1.56	0.94
Affect balance	1.13	1.44	

M, mean; Std, standard deviation; α, Cronbach’s α; SWLS, satisfaction with life scale.

Table 5 presents the correlations between study variables. Income was significantly correlated with SWLS and affect balance. Dividing income into three groups (low, medium, high), the average SWLS scores for the lowest and highest income groups were 3.56 and 5.11, respectively, indicating a difference of 1.0 standard deviation. The mean affect balance scores were 0.65 and 1.96 for the same income groups, showing a difference of 0.9 standard deviations. Income and education were moderately positively correlated. Subjective SES correlated substantially positively with Comsim and both correlated with SWLS and affect balance as expected. Social mobility correlated positively with SWLS and affect balance. Personal mastery, perceived constraints, and self-esteem were correlated with income, SWLS, and affect balance.

**TABLE 5:** Pearson’s correlation (r) between variables (*N* = 614).

Variables	1	2	3	4	5	6	7	8	9	10
1. Education		0.34**	0.31**	0.37**	0.19**	0.09*	-0.10**	0.11**	0.18*	0.10*
2. Income			0.47**	0.54**	0.34**	0.26**	-0.26**	0.24**	0.31**	0.27**
3. Subjective SES				0.67**	0.56**	0.39**	-0.42**	0.45**	0.56**	0.41**
4. Comsim					0.49**	0.49**	-0.49**	0.56**	0.64**	0.52**
5. Social mobility						0.27**	-0.33**	0.29**	0.37**	0.31**
6. Personal mastery							-0.68**	0.62**	0.58**	0.63**
7. Perceived constraints								-0.68**	-0.61**	-0.70**
8. Self-esteem									0.73**	0.78**
9. SWLS										0.66**
10. Affect balance										

*p < 0.05 (two-tailed tests); **p < 0.01 (two-tailed tests).

SWLS, satisfaction with life scale.

Table 6 displays the results of regression analyses. In Model 1, income emerged as a significant predictor of SWLS and affect balance, while education did not show a significant effect. Moving to Model 2, with the inclusion of subjective SES, subjective SES became a significant predictor of SWLS and affect balance, while the effect of income remained significant but reduced in magnitude. The addition of subjective SES led to a significant increase in R2 (Δ*F* = 179.25 and 68.79, respectively, both *p* < 0.001), indicating that subjective SES explains additional variance in SWLS and affect balance beyond the contribution of income.

**TABLE 6:** Hierarchical regressions of associations between predictors and SWB (beta-coefficients) (*N* = 614).

Predictors	Dependent variables	
	SWLS	Affect balance
**Model 1**		
Gender	0.17**	0.05
Age	0.03	0.14**
Education	0.07	0.02
Income	0.34**	0.28**
*R* ^2^	0.13	0.09
Δ*R*^2^	0.13	0.09
Δ*F*	22.47	15.48
**Model 2**		
Gender	0.12**	0.01
Age	-0.04	0.09*
Education	-0.02	-0.05
Income	0.10*	0.12*
Subjective SES	0.52**	0.36**
*R* ^2^	0.33	0.19
Δ*R*^2^	0.20	0.09
Δ*F*	179.25	68.79
**Model 3**		
Gender	0.13**	0.02
Age	-0.04	0.09*
Education	-0.09*	-0.11*
Income	-0.03	0.00
Subjective SES	0.25**	0.11*
Comsim	0.55**	0.49**
*R* ^2^	0.47	0.30
Δ*R*^2^	0.14	0.12
Δ*F*	163.29	101.9w7
**Model 4**		
Gender	0.13**	0.02
Age	-0.04	0.09*
Education	-0.10*	-0.11*
Income	-0.03	-0.01
Subjective SES	0.25**	0.09
Compsim	0.55**	0.49**
Social mobility	-0.01	0.05
*R* ^2^	0.47	0.30
Δ*R*^2^	0.00	0.00
Δ*F*	0.02	1.15

*p < 0.05, **p < 0.01.

SWLS, satisfaction with life scale.

In Model 3, when Comsim was added to the model, both Comsim and subjective SES emerged as significant predictors of SWLS and affect balance, with Comsim having a stronger influence. Income did not significantly predict SWB in these models. The inclusion of Comsim resulted in a significant increase in R2 (Δ*F* = 163.29 and 101.97, respectively, both *p* < 0.001), indicating that Comsim contributes to explaining additional variance in SWLS and affect balance beyond the effects of subjective SES.

In Model 4, social mobility was introduced as a predictor alongside subjective SES and Comsim. However, social mobility did not predict SWLS or affect balance, suggesting that perceived social mobility in relation to one’s parents does not independently contribute to SWB beyond the effects of subjective SES and Comsim.

**FIGURE 3:**
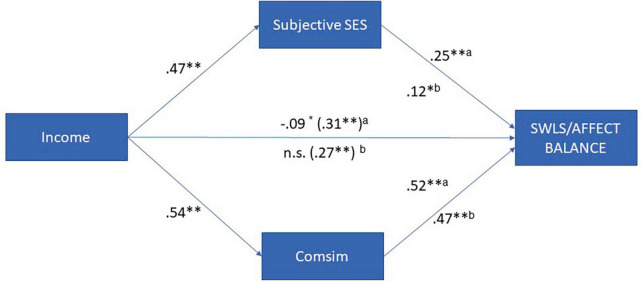
Subjective and comparative SES as mediators of the relationship between income and SWB (*N* = 614). *p ≤ 0.05; **p ≤ 0.001; ns, not significant; ^a^effects on SWLS; ^b^effects on affect balance.

To investigate the mediation hypothesis related to social comparison (hypothesis IV), we examined whether the correlations between income and SWLS, as well as affect balance, were mediated by subjective SES and Comsim. We conducted simultaneous mediation analyses including gender, age, income, and education as covariates. As depicted in Figure 3, the results revealed that the effect of income on SWLS was partially mediated by subjective SES and Comsim, with Comsim having a stronger mediating effect. Similarly, for predicting affect balance, the effect of income was fully mediated by subjective SES and Comsim, with Comsim playing a more significant role. Additional details of these mediation analyses can be found in Supplementary Table 2 (see text footnote 1).

**FIGURE 4:**
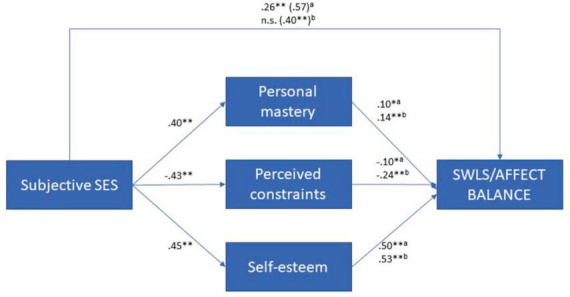
Self-perceptions as mediators between subjective SES and SWB (*N* = 614). ns, not significant; **p ≤ 0.001; *p ≤ 0.05; ^a^effects on SWLS; ^b^effects on affect balance.

**FIGURE 5:**
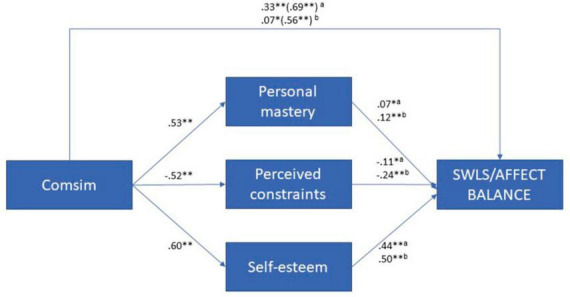
Self-perceptions as mediators between comparative SES and SWB (*N* = 614). ns, not significant; *p ≤ 0.05; **p ≤ 0.001; ^a^effects on SWLS; ^b^effects on affect balance.

Regarding hypothesis V, our findings support the partial mediation of personal mastery, perceived constraints, and self-esteem in the relationships between subjective SES and Comsim, respectively, with the SWB measures (as shown in Figures 4, 5). Notably, we observed that affective self-perceptions, represented by self-esteem, played a more prominent role in mediating these relationships compared to cognitive self-perceptions, represented by personal mastery and perceived constraints. For a comprehensive understanding of these mediation analyses, please refer to Supplementary Table 3 (see text footnote 1).

### 3.6 Interim discussion

The results of Study 2 provide further support for the findings of Study 1, reinforcing several key conclusions. Firstly, our findings confirm Study 1 findings and previous research (Tan et al., 2020) in that that income has a significant impact on SWB across the entire income range (hypothesis I). Secondly, as shown in Study 1 and in previous research (Adler et al., 2000; Operario et al., 2004; Euteneuer, 2014; Quon and McGrath, 2014; Präg et al., 2016; Demakakos et al., 2018; Tan et al., 2020; Vezzoli et al., 2022), subjective SES emerges as a predictor of SWB that is independent of income (hypothesis II). Thirdly, as in Study 1, Comsim demonstrates its unique predictive power for SWB beyond income and subjective SES (hypothesis III). Fourthly, the study confirms Study 1 in that subjective SES and Comsim jointly mediate the relationship between income and SWB (hypothesis IV). Regarding hypothesis V, our results indicate that self-perceptions play a mediating role between subjective SES and Comsim, respectively, and the measures of SWB. Interestingly, the findings suggest that self-esteem plays a more prominent role in mediating these relationships compared to self-control perceptions.

## 4 General discussion

The aim of this article was to examine the role of social comparison processes in mediating the relationship between income and SWB. The article presents two studies that investigate the relationship between income and SWB, with correlations consistent with a recent meta-analytic finding (Tan et al., 2020). Although these correlations may be moderate in strength, income remains a significant factor contributing to socioeconomic disparities in SWB. Firstly, research suggests that income likely plays a causal role in determining SWB (Dwyer and Dunn, 2022; McGuire et al., 2022; Thomson et al., 2022). Moreover, even a modest correlation between income and SWB can result in substantial differences in the SWB between individuals at the lower and upper ends of the income spectrum (Lucas and Schimmack, 2009). In our study, when income was divided into three categories, we observed a difference of approximately one standard deviation in SWB measures between the low-income group and the high-income group. Additionally, our findings align with previous research demonstrating that higher income is associated with better SWB across the income spectrum (Killingsworth, 2021; Killingsworth et al., 2023). This pattern parallels observations in health research, known as the “socioeconomic health gradient paradox,” which reflects the consistent association between improved health and incremental increases in income across the entire income distribution. This finding holds true for countries with different levels of economic development and social welfare (Adler and Stewart, 2010; Braveman et al., 2010; Huijts et al., 2010; Espelt et al., 2011; Bonaccio et al., 2016, 2020; Eikemo et al., 2016; Veronesi et al., 2016; Bray et al., 2018). In summary, our study highlights the crucial role of income in socioeconomic disparities in SWB, emphasizing the consistent association between lower income and lower SWB. It is important to note that while the correlation between income and SWB may appear weaker compared to the correlation between psychological traits and SWB, this distinction could be attributed to the differences in the constructs themselves. Psychological traits may have a stronger association with SWB due to their closer conceptual and methodological alignment, whereas income, as an external factor, may have less overlap with SWB (Hobbs and Ong, 2023).

**FIGURE 6:**
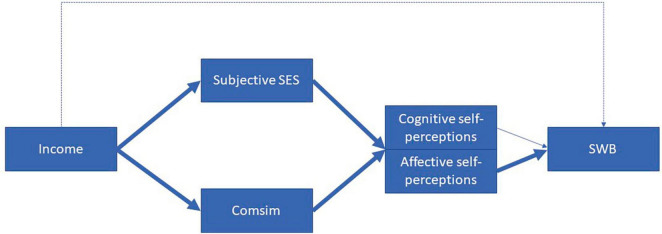
Schematic presentation of main findings.

We examined social comparison processes as mediators of the association between income and SWB. The main results are schematically presented in Figure 6. Analyses showed that the relationship between income and SWB was almost entirely mediated through social comparison processes, favoring the relativity hypothesis over the materialist explanation. This suggests that income functions primarily as a source of information for social comparisons, which in turn influence individuals’ SWB. These findings are in line with social comparison theory, which posits that individuals engage in both upward and downward social comparisons, and the outcomes of these comparisons can have varying effects on their SWB (Wood, 1989; Gibbons and Gerrard, 1991; Wills, 1991; Aspinwall and Taylor, 1993; Lyubomirsky and Ross, 1997; Suls et al., 2002; Buunk and Gibbons, 2007; Gerber et al., 2018; Crusius et al., 2022; Dufhues et al., 2023).

The results of our study are in line with previous research that has demonstrated the mediating role of subjective SES in the relationship between income and SWB (Tan et al., 2020). Subjective SES, which refers to individuals’ perception of their socioeconomic status in comparison to others in society, acts as an intermediary factor that helps explain how income influences SWB. This finding highlights the importance of considering individuals’ subjective evaluations of their social standing when examining the impact of income on their wellbeing (Tan et al., 2020; Vezzoli et al., 2022). However, our regression models and mediation analyses provide strong evidence for a unique effect of subjective SES on SWB, supporting the notion that individuals have the ability to make comparisons of themselves to others within society. The significant relationships observed in our study indicate that subjective SES plays a crucial role in shaping individuals’ wellbeing, highlighting the subjective nature of socioeconomic status and its influence on SWB.

Our study sheds light on the importance of the social comparison process of Comsim in understanding individuals’ wellbeing. The results indicate that Comsim, which involves comparing one’s current socioeconomic status to those who had a similar background during childhood, is a stronger predictor of SWB than subjective SES. This finding suggests that comparisons with similar others hold particular psychological significance and have a greater impact on individuals’ wellbeing. The regression analyses provided evidence for the predictive power of Comsim in explaining SWB, surpassing the influence of subjective SES. Moreover, the mediation analyses revealed that Comsim played a more significant mediating role in the relationship between income and SWB compared to subjective SES. These findings are in line with the similarity hypothesis of social comparison theory, which suggests that individuals tend to place greater importance on comparisons with similar others when evaluating their own wellbeing (Festinger, 1954; Buunk and Gibbons, 2007).

By highlighting the significance of Comsim in the context of socioeconomic comparisons, our study contributes to a more nuanced understanding of the mechanisms underlying the relationship between income and SWB. It underscores the need to consider the social comparison processes individuals engage in, particularly with similar others, to gain a comprehensive understanding of how socioeconomic factors influence wellbeing. One possible explanation is that Comsim captures the tendency that people compare themselves with others with whom they have had direct experience and have been closely associated with in the social system (Dufhues et al., 2023), which likely provides more diagnostic information about the self, a mechanism known as the “local dominance effect” (Zell and Alicke, 2010). Another explanation may be that Comsim better captures the impact of changes in socioeconomic status over time on SWB, while subjective SES reflects a relatively static comparison with others. Such speculation would seem to be in accordance with recent findings in health sociology research, which have highlighted the significant association between (perceived and actual) social mobility (in relation to one’s parents) and health and SWB (Euteneuer and Schäfer, 2018; Mendoza et al., 2018; Yan et al., 2018; Gugushvili et al., 2019, 2022; Euteneuer et al., 2021; Gugushvili and Präg, 2021; Präg and Gugushvili, 2021). However, although we observed a bivariate correlation between social mobility and SWB (Table 5), this association did not persist in the multivariate regression models (Table 6, Model 4). Hence, the social mobility mechanism was not supported herein.

Our study supported hypothesis V, which proposed that cognitive self-perceptions, specifically, personal control, would mediate the relationship between social comparison and SWB. Personal control refers to an individual’s belief in their ability to influence or control important life outcomes (Lachman and Weaver, 1998b; Seeman, 2008). Lower personal control has been associated with greater difficulties in controlling the present and predicting the future, as documented in several previous studies (Sherer et al., 1982; Cohen et al., 1999; Ellis et al., 2009; Kraus et al., 2009, 2012; Shah et al., 2012; Haushofer and Fehr, 2014; Farah, 2017; Ishii et al., 2017; Sapolsky, 2017; Sheehy-Skeffington and Rea, 2017). Furthermore, being positioned lower in a social system is often associated with increased unpredictability, threats, adversities, reduced social support and future opportunities, as well as reduced levels of protection, power, and popularity (Adler et al., 2000; Sapolsky, 2004, 2017; Kraus et al., 2012, 2013; Haushofer and Fehr, 2014; Sheehy-Skeffington and Haushofer, 2014; Pickett and Wilkinson, 2015; Pepper et al., 2017; Wilkinson and Pickett, 2018; Schneider, 2019; Kraft and Kraft, 2021; Galvan et al., 2022; Kraft et al., 2022). These factors may possibly contribute in explaining the positive correlation between lower control and lower SWB in our study, which is consistent with findings from several previous studies (Magaletta and Oliver, 1999; Bandura, 2001; Barlow et al., 2002; Bandura et al., 2003; Kuijer and De Ridder, 2003; Maddux and Gosselin, 2003; Santos, 2014). Together, these results suggest that personal control may play a key role in the relationship between social comparison and SWB, possibly by affecting an individual’s sense of agency, autonomy, and confidence in shaping their life outcomes.

Our study found that self-esteem played a more significant mediating role than cognitive self-perceptions (personal control) in the relationship between social comparison and SWB. This result aligns with previous research linking self-esteem to both cognitive and affective aspects of wellbeing. Individuals with high self-esteem tend to report higher levels of life satisfaction, positive affect, and overall wellbeing (Diener and Diener, 1995; Seligman and Csikszentmihalyi, 2000; Robins et al., 2001; Baumeister and Vohs, 2003; Tesser, 2003; Diener et al., 2015). One possible explanation for our finding is that adults perceive socioeconomic status as an earned status (Tesser, 1988, 2003; Lachman and Weaver, 1998b; Suls et al., 2002; Sapolsky, 2017), where an individual’s perceived abilities and accomplishments in comparison to similar others are crucial for their sense of self-worth. In this context, self-esteem becomes a key factor in the translation of income comparisons into overall wellbeing. The importance of self-worth suggests that individuals’ positive evaluation of themselves and their accomplishments can contribute significantly to their SWB.

Another potential explanation is that there may be more conceptual and methodological overlap between the measures of social comparison and self-esteem compared to social comparison and personal control. This overlap may have led to self-esteem emerging as a stronger mediator in our study. Further research could delve into the relationships between these constructs and their respective measurement tools to gain a more comprehensive understanding.

By highlighting the critical role of self-worth in the relationship between income and SWB, our study adds valuable insights to the existing literature. It underscores the significance of individuals’ self-perception and self-evaluation in shaping their wellbeing outcomes. Future research can further explore the mechanisms and processes through which self-esteem influences the relationship between social comparison, income, and SWB, providing a deeper understanding of the psychological factors at play.

## 5 Limitations

Several limitations should be acknowledged regarding this study. Firstly, while we collected data on predictors and SWB at different time points, we did not measure changes in these variables over time. One reason for this is that substantial changes in SES variables over short time intervals are unlikely, although social mobility can occur over longer periods. Therefore, we were unable to establish causal or temporal inferences using fixed effect or cross-lagged panel models (Maxwell and Cole, 2007; Hamaker et al., 2015). We welcome future research endeavors that employ long-term longitudinal designs, as they are better suited for investigating causal or temporal inferences. These studies can provide valuable insights into the dynamics of variables over extended periods, offering a more comprehensive understanding of the complex relationships. Secondly, it’s important to consider that self-reported data may be subject to social desirability and recall bias, which could impact the validity of the results (Johnson and Fornell, 1991; Althubaiti, 2016). Thirdly, it’s worth noting that measures of perceived socioeconomic rank and SWB may be subject to common method variance, reporting bias, or influenced by shared traits such as negative affectivity (Spector, 1987; Watson et al., 1988; Podsakoff et al., 2012).

## 6 Implications

These findings may have significant clinical and political implications. Mental health professionals should be aware of the impact of social comparison processes on individuals’ SWB and consider these factors in their assessments and treatment plans. For instance, therapists could use cognitive-behavioral therapy to help clients develop more positive and realistic self-perceptions, including their perceived socioeconomic rank relative to similar others. Additionally, interventions aimed at enhancing SWB should consider the role of social comparison processes and provide tools and strategies to manage them. From a political perspective, this study emphasizes the need for policies that address the impact of social comparison processes on individuals’ wellbeing, especially in the context of income inequality. Policies aimed at reducing income inequality may help mitigate the negative impact of social comparison processes on SWB, as more individuals may engage in less socioeconomically unfavorable comparisons. Furthermore, policies that provide support and resources for individuals in lower socioeconomic positions could also improve their SWB by reducing stress and increasing feelings of control and predictability.

## 7 Conclusion

In conclusion, this study provides evidence for the mediating roles of subjective SES, Comsim, and self-perceptions in the relationship between income and SWB. The results suggest that income is not only directly associated with SWB but also operates through social comparison processes and individual self-perceptions. Subjective SES and Comsim play crucial roles in explaining how income influences SWB, with Comsim emerging as a particularly strong mediator. Moreover, our study highlights the importance of self-esteem in mediating the relationship between social comparison and SWB, suggesting that individuals’ self-worth plays a significant role in translating income comparisons into overall wellbeing. These findings enhance our understanding of the complex mechanisms underlying the income-SWB association and emphasize the need to consider multiple psychological factors in future research and interventions aimed at promoting wellbeing.

## Data availability statement

The datasets presented in this study can be found in online repositories. The names of the repository/repositories and accession number(s) can be found below: https://osf.io/hy98r/.

## Ethics statement

The studies involving humans were approved by the Ethics Committee, Department of Psychology, University of Oslo. The studies were conducted in accordance with the local legislation and institutional requirements. The participants provided their written informed consent to participate in this study.

## Author contributions

PK: Conceptualization, Data curation, Formal analysis, Investigation, Methodology, Project administration, Writing—original draft, Writing—review and editing. BK: Conceptualization, Data curation, Formal analysis, Methodology, Writing—original draft, Writing—review and editing.

## Footnotes

1
https://osf.io/xqcne/
