# Supplementary material for: The income-happiness nexus: uncovering the importance of social comparison processes in subjective wellbeing
Source: Front Psychol. 2023 Nov 23;14:1283601. doi: 10.3389/fpsyg.2023.1283601 (PMC10702233; doi:10.3389/fpsyg.2023.1283601)
Supplement: Supplementary file 1 [file Data_Sheet_1.pdf]

*Supplementary Material*

# The Income-Happiness Nexus: Uncovering the Importance of Social Comparison Processes in Subjective Well-Being.

**Pål Kraft*, Brage Kraft**

*** Correspondence:** pal.kraft@psykologi.uio.no

## Data availalability

The data supporting the conclusions of this article are accessible on OSF (Open Science Framework); https://osf.io/xqcne/

## Description of the sampling procedure in Prolific.

Prolific is an online platform commonly used by researchers to recruit participants for studies. The sampling process in Prolific typically follows these steps:

Registration: Participants interested in participating in research studies on Prolific must first register on the platform. During registration, they provide basic demographic information such as age, gender, location, and other relevant details.

Account Verification: Prolific verifies participant accounts to ensure the authenticity of their information. This verification process may involve confirming participants' email addresses, phone numbers, or even government-issued IDs in some cases.

Eligibility Screening: Researchers create studies on Prolific and specify the criteria for participant eligibility. Participants are matched with studies based on their demographic information and the eligibility criteria set by researchers.

Study Listings: Studies conducted by researchers are listed on the Prolific platform. Participants can browse through available studies and choose to participate in those for which they meet the eligibility criteria.

Participant Selection: Participants self-select into studies by clicking on the study listings that interest them. Researchers can also choose to pre-screen participants based on specific criteria before they are allowed to participate.

Informed Consent: Before participating in a study, participants are presented with information about the study, including its purpose, procedures, and any potential risks or benefits. They must provide wrtitten informed consent before proceeding.

Data Collection: Participants complete the study tasks or surveys as instructed by the researcher. We collected data through Qualtrics, an online questionnaire system.

Compensation: Participants are compensated for their time and effort. Compensation was done in the form of cash payments according to Prolific's schedule. Prolific handled the payment process.

Data Delivery: Once participants complete a study, researchers can access the collected data through the Prolific platform. Data were be downloaded for further analysis.

Review and Payment: Researchers review the data and validate participants' completion of the study. After verification, they approve payments to participants.

Participant Feedback: Participants were given the option to leave feedback about their experience with the study and the researcher. This feedback can help maintain the quality of research conducted on the platform.

Prolific's sampling process is designed to provide researchers with a pool of diverse and motivated participants for their studies while ensuring data quality and participant compensation (Palan & Schitter, 2018; Peer et al., 2017).

**Comparing participants who participated in the T1-study only and those who participated in both the T1 and T2-studies.**

Study 1

A two sample independent t-test was performed to compare gender, age, education, income and subjectice SES between those who participated in both data collections (T1 & T2; N = 588) and those who participated in the first collection only (T1; n = 91). There was not a significant difference in gender between T1 participants (M = 1.51, SD = .50) and T1&T2 participants (M = 1.50, SD = .50; t(677) = -.10, p = .92).There was not a significant difference in age between T1 participants (M = 40.50, SD = 9.35) and T1&T2 participants (M = 41.53, SD = 9.93; t(677) = .94, p = .33). There was not a significant difference in education between T1 participants (M = 4.37, SD = 1.31) and T1&T2 participants (M = 4.24, SD = 1.39; t(677) = -.84, p = .40). There was not a significant difference in income between T1 participants (M = 2.93, SD = 1.62) and T1&T2 participants (M = 2.93, SD = 1.71; t(677) = -.04, p = .97). There was not a significant difference in subjective SES between T1 participants (M = 5.14 SD = 1.61) and T1&T2 participants (M = 5.21, SD = 1.65; t(677) = .36, p = .72). There was not a significant difference in Comsim between T1 participants (M = 4.40 SD = .85) and T1&T2 participants (M = 4.38, SD = 1.43; t(677) =-.16, p = .87).

Study 2

A two sample independent t-test was performed to compare age, gender, education, income and subjectice SES between those who participated in both data collections (T1 & T2; N = 614) and those who participated in the first collection only (T1; n = 107). There was not a significant difference in gender between T1 participants (M = 1.53, SD = .50) and T1&T2 participants (M = 1.49, SD = .50;

t(719) = .78, p = .44).There was not a significant difference in age between T1 participants (M = 43.71, SD = 11.96) and T1&T2 participants (M = 43.53, SD = 10.79; t(719) = .16, p = .88). There was not a significant difference in education between T1 participants (M = 3.33, SD = 1.28) and T1&T2 participants (M = 3.43, SD = 1.32; t(719) = -.73, p = .47). There was not a significant difference in income between T1 participants (M = 2.93, SD = 1.45) and T1&T2 participants (M = 3.08, SD = 1.50; t(719) = -1.01, p = .31). There was not a significant difference in subjective SES between T1 participants (M = 5.20 SD = 1.72) and T1&T2 participants (M = 5.21, SD = 1.72; t(719) = -.10, p = .92). There was not a significant difference in Comsim between T1 participants (M = 4.29 SD = 1.23) and T1&T2 participants (M = 4.33, SD = 1.28; t(719) = -.32, p = .75).

**Comsim – Study 1.**

In Study 1, we developed a Comsim scale to assess individuals' self-perceived socioeconomic status relative to others with similar socioeconomic backgrounds.

Participants were first presented to an introductory text, then they responded to four items:

*People come from different social classes or socioeconomic backgrounds.This reflects how much money your parents had; how much education your parents had; your parent's social position; your housing conditions when you were a child; the schools you attended; your place of living as a child. When responding to the statements below, think about such elements in your socioeconomic background, that is, where you come from socioeconomically:*

*Compared to other people coming from a similar socioeconomic background*

*(1) My current financial situation is quite good*

*(2) My current educational situation is quite good*

*(3) I have been quite successful in work-life*

*(4) I think my current socioeconomic position is quite good*

The items were responded to on a 7-point scale ranging from strongly disagree (1) to strongly agree (7).

Statistics

A principal component factor analysis (varimax rotation) was performed on the four items and produced a one-factor solution, which explained 78.9% of the inter-item variance (Eigenvalue = 3.15), with α = .91. The correlation between this Comsim scale and income was .40 (p<.001).

**Comsim – Study 2.**

In Study 2, we developed an alternative measure of Comsim. As in Study 1, we wanted to assess individuals' self-perceived socioeconomic status relative to others with similar socioeconomic backgrounds. However, in this measure we wanted the participants to compare themselves specifically to childhood friends and schoolmates in order to capture the tendency that people have to compare themselves with others with whom they have had direct experience and have been closely associated with in the social system (Dufhues et al., 2023; Zell & Alicke, 2010). We anticipated that such friends and schoolmates would generally come from a similar socioeconomic background.

Participants were first presented to an introductory text, then they responded to four items:

*Think about the friends and schoolmates you had when you were a child. How do you think you have done in life, when you compare yourself to them?*

*Compared to my childhood friends and schoolmates:*

(1) *My education is quite good*
(2) *My work-life has been quite successful*
(3) *My income is quite good*
(4) *My social status is quite high*

The items were responded to on a 7-point scale ranging from strongly disagree (1) to strongly agree (7).

<u>Statistics</u>

A principal component factor analysis (varimax rotation) produced a one-factor solution that explained 70.6% of the inter-item variance (Eigenvalue = 2.28), with with $\alpha = .86$. The correlation between this Comsim scale and income was .49 ($p<.001$).

**Social mobility – Study 2**

In Study 2, social mobility in relation to one's parents was assessed using a modified version of the MacArthur Scale of Subjective Social Status - Adult Version (Adler et al., 1994). The following procedure was employed:

(a) First, participants were asked to indicate the social status of their father when he was the same age as the participant. The respondent could chose a number between 1-10.

*This question is about your father- when he was as old as you are now. Think of a ladder (see image) as representing where people stand in society. At the top of the ladder are the people who are best off—those who have the most money, most education and the best jobs. At the bottom are the people who are worst off—who have the least money, least education and the worst jobs or no job. The higher up one is on this ladder, the closer one is to people at the very top and the lower one is, the closer one is to the bottom. Think about your father, when he was at the same age as you are now. Where would you put him on the ladder? Choose the number whose position best represents where you put him on this ladder.*

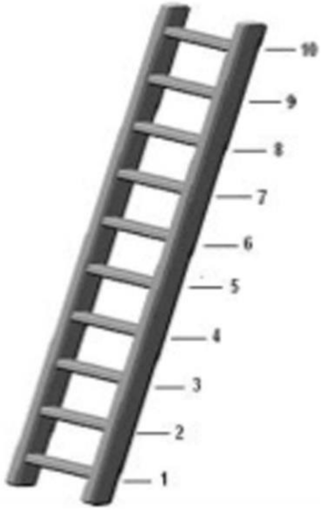


(b) Next, participants were asked to indicate the social status of their mother when she was the same age as the participant. The respondent could chose a number between 1-10.

*This question is about your mother- when she was as old as you are now. Think of a ladder (see image) as representing where people stand in society. At the top of the ladder are the people who are best off—those who have the most money, most education and the best jobs. At the bottom are the people who are worst off—who have the least money, least education and the worst jobs or no job. The higher up one is on this ladder, the closer one is to people at the very top and the lower one is, the closer one is to the bottom. Think about your mother, when she was at the same age as you are now. Where would you put her on the ladder? Choose the number whose position best represents where you put her on this ladder.*

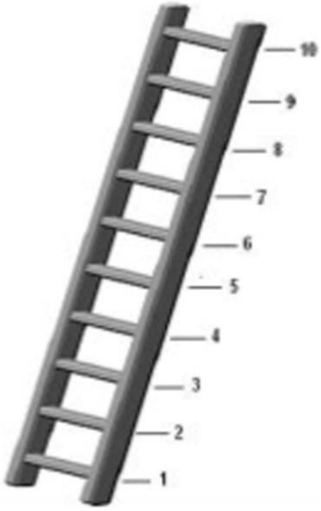


(c) Finally, participants were asked to indicate their own relative social status. Again, the respondent could chose a number between 1-10.

*Think of a ladder (see image) as representing where people stand in society. At the top of the ladder are the people who are best off—those who have the most money, most education and the best jobs. At the bottom are the people who are worst off—who have the least money, least education and the worst jobs or no job. The higher up you are on this ladder, the closer you are to people at the very top and the lower you are, the closer you are to the bottom. Where would you put yourself on the ladder? Choose the number whose position best represents where you are on this ladder.*

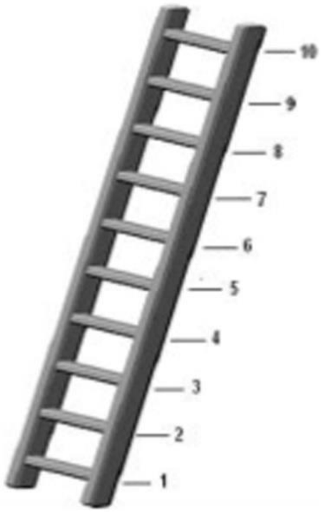


Then, the following calculation was performed: An average of fathers (a) and mothers (b) social status was calculated. This average value was next subtracted from the respondents own social status score (c), to arrive at a value for the participants social mobility vis-a-vis their parents. This value could vary between -9 and + 9. A positive score indicated that the participant had experienced a positive social mobility vis-a-vis parents, while a negative score indicated a negative social mobility vis-a-vis parents.

Descriptive statistics showed that the values on the social mobility scale varied between -4.50 and +7.00; the mean was 0.29 with a standard deviation of 1.69. Skewness was .098 and kurtosis was .197.

## On the correlation between subjective SES and Comsim

If two highly correlated variables are included in a regression model together, it can sometimes lead to a situation where one variable suppresses the other's predictive power. In other words, the presence of both variables can make their unique contributions to explaining the dependent variable less clear or even contradictory.

As regards subjective SES and Comsim in predicting SWLS, this wasexamined by calculating Tolerance and Variance Inflation Factor (VIF) for each predictor in a multiple regression analysis.

Generally, tolerance values close to 1 indicate low colliearity, and generally values above 0.1 are considered acceptable. As regards VIF, values above 10 or 5 (some use a stricter threshold of 2.5) are often indicative of collinearity.

In our study 1 data, when subjective SES and Comsim were entered as predictors of SWLS, for subjective SES and Comsim the tolerance indicator was .69 and the VIF was 1.48. In study 2 data, these indicators were .56 and 1.80, respectively. This indicates that we have low or no collinearity concerns.

Additionally, if the predictors are substantially correlated, one should consider conducting separate analyses with each predictor in the model while controlling for the other. This can help us understand their unique contributions to the dependent variable by partialling out shared variance. To investigate this possibility, we ran regression analyses with Comsim and Subjective SES both separately and together in the model, since this would allow us to assess whether their inclusion together changes the coefficients or significance levels of these variables and if any suppressor effect is evident.

We then looked for changes in coefficients and significance levels between the separate and combined analyses. If the inclusion of both variables together substantially changed their coefficients or significance levels compared to when they are analyzed separately, it could indicate a suppressor effect.

In study 1, the correlation between subjective SES and Comsim was .56 ($p<.001$). Moreover, both were positively correlated with SWLS (.46 and .64, respectively, both $p<.001$). In the regression analysis, $R^2$ was .43, which is below .the value of .50, above where one would suspect supression to take place. Furthermore, in the full regression models, both predictors had significant positive beta-coefficients predicting SWLS; i.e. none of them were negative. Furthermore, the sum of the squared partial correlation coefficients was .23. This is lower than the $R^2$ (.43) in the regression model, which indicates that there was no supression.

As regards study 2, the correlation between subjective SES and Comsim was .67 ($p<.001$). Both subjective SES and Comsim were positively correlated with SWLS (.56 and .64, respectively, both $p<.001$). In the regression analysis, $R^2$ was .44, which is again was below the value of .50, above which one would suspect supression to take place. Furthermore, in the full regression models, both predictors had significant beta-coefficients predicting SWLS; i.e. none of them are negative. Furthermore, the sum of the squared partial correlation coefficients was .16. This is lower than the $R^2$ (.44) in the regression model. This indicates that there was no supression.

**Table S1.** PROCESS macro mediation analyses: Subjective SES and Comsim as mediators between income and SWB. Standardised regression coefficients based on 5000 bootstrap samples. Sex, age and education were included as co-variates in all models (N = 588).

| **Study 1 (N = 588)** | | | | | |
|---|---|---|---|---|---|
| **Predicting CES-D** | *b* | *SE* | *t* | *p* | *95% CI* |
| Income ---> Subjective SES | .44 | .04 | 11.78 | .001 | .37-.51 |
| Income ---> Comsim | .40 | .04 | 10.64 | .001 | .33 -.48 |
| Subjective SES ---> CES-D | -.16 | .05 | -3.45 | .001 | -.25 - -.07 |
| Comsim ---> CES-D | -.35 | .05 | -7.62 | .001 | -.44- -.26 |
| Direct effect of income ---> CES-D | .02 | .04 | .38 | .71 | -.07 - .10 |
| | *Effect* | *BootSE* | *BootLLCI* | *BootULCI* | |
| Total indirect effect of income | -.21 | .03 | -.26 | -.16 | |
| Indirect effect via Subjective SES* | -.07 | .02 | -.12 | -.03 | |
| Indirect effect via Comsim | -.14 | .02 | -.19 | -.10 | |
| **Predicting SWLS** | *b* | *SE* | *t* | *p* | *95% CI* |
| Income ---> Subjective SES | .44 | .04 | 11.78 | .001 | .37 - .51 |
| Income ---> Comsim | .40 | .04 | 10.64 | .001 | .33 - .48 |
| Subjective SES ---> SWLS | .15 | .04 | 3.86 | .001 | .07 - .23 |

| | | | | | |
|---|---|---|---|---|---|
| Comsim ---> SWLS | .58 | .04 | 15.16 | .001 | .51 - .66 |
| Direct effect of income --->SWLS | -.05 | .04 | -1.38 | .17 | -.12 - .02 |
| | *Effect* | *BootSE* | *BootLLCI* | *BootULCI* | |
| Total indirect effect of income | .30 | .03 | .25 | .36 | |
| Indirect effect via Subjective SES* | .07 | .02 | .03 | .10 | |
| Indirect effect via Comsim | .23 | .03 | .19 | .29 | |
| **Study 2 (N = 614)** | | | | | |
| **Predicting SWLS** | *b* | *SE* | *t* | *p* | *95% CI* |
| Income ---> Subjective SES | .47 | .04 | 13.32 | .001 | .40 - .54 |
| Income ---> Comsim | .54 | .03 | 15.67 | .000 | .47 - .60 |
| Subjective SES ---> SWLS | .25 | .04 | 6.10 | .000 | .17 - .33 |
| Comsim ---> SWLS | .52 | .04 | 12.24 | .000 | .44 - .61 |
| Direct effect of income | -.09 | .04 | -.2.47 | .01 | -.16 - .02 |
| | *Effect* | *BootSE* | *BootLLCI* | *BootULCI* | |
| Total indirect effect of income | .40 | .03 | .35 | .45 | |
| Indirect effect via Subjective SES* | .12 | .02 | .08 | .16 | |
| Indirect effect via Comsim | .28 | .03 | .23 | .34 | |

| **Predicting affect balance** | *b* | *SE* | *t* | *p* | *95% CI* |
|---|---|---|---|---|---|
| Income ---> Subjective SES | .47 | .04 | 13.32 | .000 | .40 - .54 |
| Income ---> Comsim | .54 | .03 | 15.67 | .000 | .47 - .56 |
| Subjective SES ---> affect balance | .12 | .05 | 2.46 | .014 | .02 - .21 |
| Comsim ---> affect balance | .47 | .05 | 9.59 | .000 | .37 - .56 |
| Direct effect of income | -.04 | .04 | -.92 | .360 | -.12 - .04 |
| | *Effect* | *BootSE* | *BootLLCI* | *BootULCI* | |
| Total indirect effect of income | .31 | .03 | .25 | .37 | |
| Indirect effect via Subjective SES* | .06 | .02 | .01 | .10 | |
| Indirect effect via Comsim | .25 | .03 | .19 | .31 | |

*Boots SE and CI (lower and upper levels)

SWLS = satisfaction with life scale

CES-D: Center for epideiological studies depression scale

**Table S2.** Self-perceptions as mediators of the relationship between Subjective SES and and SWB. PROCESS macro mediation analyses. Standardized regression coefficients based on 5000 bootstrap samples. Sex, age and education were included as co-variates in all models. Study 2 (N = 614).

| | | | | | |
|---|---|---|---|---|---|
| **Predicting SWLS** | *b* | *SE* | *t* | *p* | *95% CI* |
| Subjective SES ---> Personal mastery | .40 | .04 | 10.20 | .000 | .33 - .48 |
| Subjective SES ---> Perceived constraints | -.43 | .04 | -11.11 | .000 | -.50 -- .36 |
| Subjective SES ---> Self-esteem | .45 | .04 | 11.80 | .000 | .03 -.17 |
| Personal mastery ---> SWLS | .10 | .04 | 2.80 | .01 | .44 - .61 |
| Peceived constraints ---> SWLS | -.10 | .04 | -.2.56 | .01 | -.17 - .02 |
| Self-esteem ---> SWLS | .50 | .04 | 13.38 | .000 | .42 - .57 |
| | *Effect* | *BootSE* | *BootLLCI* | *BootULCI* | |
| Total indirect effect of Subjective SES on SWLS | .30 | .03 | .25 | .36 | |
| Direct effect of Subjective SES on SWLS | .26 | .03 | .21 | .33 | |
| Indirect effect via Personal mastery on SWLS | .04 | .02 | .01 | .07 | |
| Indirect effect via Perceived constraints on SWLS | .04 | .02 | .01 | .08 | |
| Indirect effect via Self-esteem on SWLS | .22 | .03 | .17 | .28 | |
| | | | | | |
| **Predicting affect balance** | *b* | *SE* | *t* | *p* | *95% CI* |

| | | | | | |
|---|---|---|---|---|---|
| Subjective SES ---> Personal mastery | .40 | .04 | 10.20 | .000 | .33 - .48 |
| Subjective SES ---> Perceived constraints | -.43 | .04 | -11.11 | .000 | -.50 -- .36 |
| Subjective SES ---> Self-esteem | .45 | .04 | 11.80 | .000 | .03 -.17 |
| Personal mastery ---> Affect balance | .14 | .03 | 4.06 | .000 | .07 -. 20 |
| Peceived constraints ---> Affect balance | -.24 | .04 | -6.75 | .000 | -.31 - .-17 |
| Self-esteem ---> Affect balance | .53 | .04 | 15.08 | .000 | .46 - .59 |
| | *Effect* | *BootSE* | *BootLLCI* | *BootULCI* | |
| Total indirect effect of Subjective SES on Affect balance | *.39* | *.03* | *.33* | *.46* | |
| Direct effect of Subjective SES on Affect balance | .01 | .03 | -.04 | .07 | |
| Indirect effect via Personal mastery on Affect balance | .06 | .02 | .03 | .09 | |
| Indirect effect via Perceived constraints on Affect balance | .10 | .02 | .07 | .15 | |
| Indirect effect via Self-esteem on Affect balance | .23 | .03 | .19 | .29 | |

*Boots SE and CI (lower and upper levels)

SWLS = satisfaction with life scale

**Table S3.** Self-perceptions as mediators of the relationship between Comsim and and SWB. PROCESS macro mediation analyses. Standardised regression coefficients based on 5000 bootstrap samples. Sex, age and education were included as co-variates in all models. Study 2 (N = 614).

| | | | | | |
|---|---|---|---|---|---|
| **Predicting SWLS** | *b* | *SE* | *t* | *p* | *95% CI* |
| Comsim ---> Personal mastery | .53 | .04 | 14.00 | .000 | .46 - .61 |
| Comsim ---> Perceived constraints | -.52 | .04 | -13.63 | .000 | -.59 - - .44 |
| Comsim ---> Self-esteem | .60 | .04 | 17.10 | .000 | .53 - .67 |
| Personal mastery ---> SWLS | .07 | .04 | 2.01 | .05 | .01 - .14 |
| Peceived constraints ---> SWLS | -.11 | .04 | -.2.97 | .01 | -.18 - - .04 |
| Self-esteem ---> SWLS | .44 | .04 | 11.57 | .000 | .06 - .15 |
| | *Effect* | *BootSE* | *BootLLCI* | *BootULCI* | |
| Total indirect effect of Comsim on SWLS | .36 | .03 | .30 | .41 | |
| Direct effect of Comsim on SWLS | .33 | .03 | .26 | .39 | |
| Indirect effect via Personal mastery on SWLS | .04 | .02 | .00 | .08 | |
| Indirect effect via Perceived constraints on SWLS | .06 | .02 | .02 | .11 | |
| Indirect effect via Self-esteem on SWLS | .26 | .03 | .21 | .32 | |
| | | | | | |
| **Predicting affect balance** | *b* | *SE* | *t* | *p* | *95% CI* |

| | | | | | |
|---|---|---|---|---|---|
| Comsim ---> Personal mastery | .53 | .04 | 13.32 | .000 | .46 - .61 |
| Comsim ---> Perceived constraints | -.52 | .03 | -13.63 | .000 | -.59 - -44 |
| Comsim ---> Self-esteem | .60 | .04 | 17.07 | .000 | .53 - .67 |
| Personal mastery ---> Affect balance | .12 | .03 | 4.06 | .000 | .07 -. 20 |
| Peceived constraints ---> Affect balance | -.24 | .04 | -6.70 | .000 | -.31 - -.17 |
| Self-esteem ---> Affect balance | .50 | .04 | 14.01 | .000 | .43 - .57 |
| | *Effect* | *BootSE* | *BootLLCI* | *BootULCI* | |
| Total indirect effect of Comsim on Affect Balance | .49 | .03 | .43 | .56 | |
| Direct effect of Comsim on Affect Balance | .07 | .03 | .00 | .13 | |
| Indirect effect via Personal mastery on Affect Balance | .07 | .02 | .03 | .11 | |
| Indirect effect via Perceived constraints on Affect Balance | .12 | .02 | .08 | .17 | |
| Indirect effect via Self-esteem on Affect Balance | .30 | .03 | .25 | .36 | |

*Boots SE and CI (lower and upper levels)

SWLS = satisfaction with life scale

**References**


Dufhues, T., Möllers, J., Jantsch, A., Buchenrieder, G., & Camfield, L. (2023). Don't Look Up! Individual Income Comparisons and Subjective Well-Being of Students in Thailand. *Journal of Happiness Studies*, *24*(2), 477-503. https://doi.org/10.1007/s10902-022-00604-4

Palan, S., & Schitter, C. (2018). Prolific.ac—A subject pool for online experiments. *Journal of Behavioral and Experimental Finance*, *17*, 22-27. https://doi.org/10.1016/j.jbef.2017.12.004

Peer, E., Brandimarte, L., Samat, S., & Acquisti, A. (2017). Beyond the Turk: Alternative platforms for crowdsourcing behavioral research. *Journal of Experimental Social Psychology*, *70*, 153-163. https://doi.org/10.1016/j.jesp.2017.01.006

Zell, E., & Alicke, M. (2010). The Local Dominance Effect in Self-Evaluation: Evidence and Explanations. *Personality and social psychology review : an official journal of the Society for Personality and Social Psychology, Inc*, *14*, 368-384. https://doi.org/10.1177/1088868310366144
